# Global Research Trends in Home Mechanical Ventilation: A Bibliometric Analysis

**DOI:** 10.3390/healthcare14111578

**Published:** 2026-06-04

**Authors:** Ferhan Demirer Aydemir, Volkan Hanci

**Affiliations:** 1 Department of Intensive Care Medicine, Faculty of Medicine, Canakkale Onsekiz Mart University, 17100 Canakkale, Turkey; 2 Department of Anesthesiology and Reanimation, Faculty of Medicine, Izmir Dokuz Eylul University, 35340 Izmır, Turkey; vhanci@gmail.com

**Keywords:** home mechanical ventilation, bibliometric analysis, citation analysis, Web of Science, research trends

## Abstract

**Highlights:**

**What are the main findings?**

- Exploratory associations were observed between citation metrics and journal quartile/SCI-Expanded index status in home mechanical ventilation research.
- Publications after 2020 showed the highest citations per year, whereas studies from 2005 to 2009 had the highest total citations.

**What are the implications of the main findings?**

- Publishing in higher-quartile and SCI-Expanded journals may improve scientific visibility in home mechanical ventilation research.
- Identified citation trends may guide future research priorities and healthcare planning in long-term ventilatory care.

**Abstract:**

Background/Objectives: Home mechanical ventilation (HMV) has become an essential component of long-term respiratory support for patients with chronic respiratory failure. Despite the growing number of publications, the characteristics and citation patterns of the most influential studies have not been systematically evaluated. This study aimed to analyze the 50 most-cited publications on home mechanical ventilation indexed in the Web of Science Core Collection and explore citation patterns and potential associations with citation impact. Methods: This study was designed as a descriptive citation-based bibliometric analysis. A bibliometric analysis was performed using the Web of Science Core Collection. Publications related to home mechanical ventilation were ranked by total citation count, and the 50 most-cited articles were included. Extracted variables included publication year, total citations, citations per year, journal quartile, impact factor, index status, article type, topic category, geographic origin, and ventilation population category. Descriptive statistics were calculated. Group comparisons were performed using the Kruskal–Wallis and Mann–Whitney U tests, and correlations were evaluated with Spearman’s analysis. Funding status was summarized descriptively because funding was reported in only 8 studies. Results: The median total citation count was 15.5 (range: 1–118), and the median citations per year was 0.89 (range: 0.02–9.83). Most articles were published in Q1 journals and indexed in SCI-Expanded. Exploratory associations were observed between citation metrics and journal quartile/index status (*p* < 0.05). Articles published between 2005 and 2009 had the highest total citations, whereas those published after 2020 showed the highest citations per year. No association was observed with geographic origin. Conclusions: Distinct exploratory citation patterns were observed according to publication period, journal quartile, and index status. Bibliometric evaluation may help characterize the academic development and visibility of home mechanical ventilation research, but these findings should not be interpreted as confirmatory determinants of citation impact.

## 1. Introduction

Bibliometric analysis has become an established methodological approach for examining the intellectual structure and scientific influence of a research field [1]. By quantifying citation performance and publication characteristics, bibliometric studies allow for the identification of influential publications, emerging themes, and structural patterns in academic production [2]. Analyses focusing on the “most cited” articles are particularly informative, as citation counts are commonly used as a proxy for scholarly impact and knowledge dissemination [3]. Although citations do not directly equate to methodological quality, highly cited publications often reflect topics that have shaped clinical practice, informed guidelines, or redirected research priorities [4].

Home mechanical ventilation (HMV) represents a rapidly evolving domain within respiratory and critical care medicine [5]. Advances in ventilator technology, monitoring systems, and multidisciplinary home care models have enabled long-term ventilatory support outside the hospital environment [6]. HMV is increasingly used for patients with neuromuscular disorders, chronic obstructive pulmonary disease, chest wall diseases, and other causes of chronic respiratory failure [7]. Beyond technical considerations, the field encompasses survival outcomes, quality of life, caregiver burden, ethical decision-making, and healthcare system organization [8]. As the population of ventilator-assisted individuals grows, the scientific literature addressing these dimensions has expanded accordingly [9].

Despite the clinical and organizational relevance of HMV, the citation landscape of this field has not been systematically characterized. Understanding which studies have exerted the greatest influence may provide insight into the evolution of research priorities and highlight dominant publication venues. Moreover, evaluating exploratory relationships between citation metrics and journal characteristics—such as quartile (Q index), impact factor, index status, and open access—may contribute to a more nuanced descriptive understanding of visibility in this specialty.

The present study therefore aimed to conduct a comprehensive bibliometric analysis of the 50 most-cited publications on home mechanical ventilation indexed in the Web of Science. Specifically, we sought to (1) describe their citation patterns, (2) analyze differences in total citations and citations per year across predefined subgroups, (3) evaluate correlations between citation metrics and bibliometric characteristics, and (4) identify structural trends in topic distribution, journal characteristics, and geographic authorship.

## 2. Materials and Methods

### 2.1. Study Design

This investigation was designed as a cross-sectional bibliometric analysis of the 50 most-cited publications in the field of HMV. The study focused exclusively on citation performance and publication characteristics and did not involve human participants or patient-level data.

### 2.2. Data Source and Search Strategy

The Web of Science (WoS) Core Collection database was searched using a predefined search strategy developed for home mechanical ventilation (HMV)-related publications. The searched indexes within the Web of Science Core Collection included SCI-Expanded, ESCI, Conference Proceedings Citation Index, and Book Citation Index when available. The following search query was used within the WoS advanced search interface: TS = (“home mechanical ventilation” OR “domiciliary mechanical ventilation” OR “home ventilator*” OR “long-term mechanical ventilation” OR “home respiratory support” OR “ventilator-assisted living”). Boolean operators (OR) and truncation symbols (*) were applied to increase search sensitivity and capture variations in terminology. No restrictions regarding publication year, language, or study type were initially applied. The final database search was performed on 1 February 2026.

**Figure 1:**
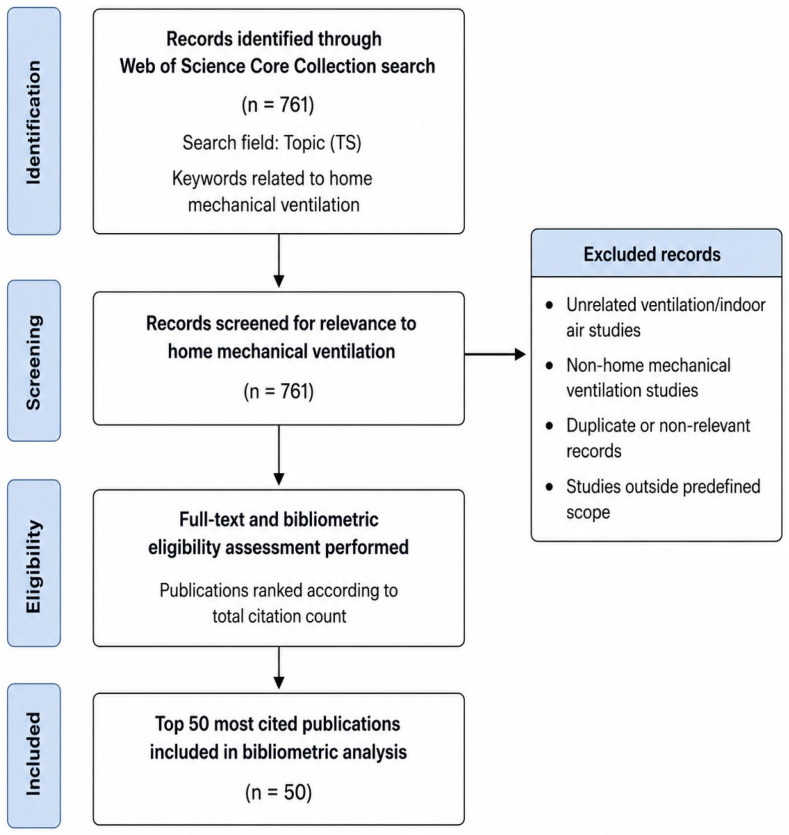
Flow diagram of the study selection process.

All retrieved records were screened manually for relevance to home mechanical ventilation. Publications unrelated to long-term home ventilatory support, duplicate records, or studies outside the predefined scope were excluded. Publications with ambiguous relevance were evaluated independently by both authors and included only after consensus was achieved. A total of 761 records were initially identified through the Web of Science search strategy and screened for relevance to home mechanical ventilation. After exclusion of duplicate, non-relevant, and out-of-scope publications, the remaining eligible records were ranked according to total citation count in descending order, and the 50 most-cited publications were included in the final bibliometric analysis. The search strategy was intentionally broad to capture the spectrum of long-term home mechanical ventilation literature; however, eligibility assessment prioritized studies involving invasive home mechanical ventilation (IMV), tracheostomy ventilation, or mixed ventilator-assisted populations in which IMV was explicitly included or clinically relevant to the study question, population, service model, or technical issue. Studies focusing exclusively on non-invasive ventilation without a direct link to invasive HMV, transition to IMV, ventilator-assisted home care, or mixed HMV service delivery were excluded. To improve transparency, each included publication was additionally categorized as IMV-only, NIV-only, or mixed/unspecified IMV/NIV in Table 1. The study selection and screening process are summarized in Figure 1.

The figure summarizes the identification, screening, eligibility assessment, and final inclusion process used to identify the 50 most-cited publications on home mechanical ventilation from the Web of Science Core Collection database.

The Web of Science Core Collection database was selected as the sole bibliographic source because it provides standardized citation indexing, structured citation tracking, journal quartile classifications, and impact factor metrics that are widely used in bibliometric research methodology. The use of a single database ensured methodological consistency and minimized variability in citation counts and journal indexing across different bibliographic platforms. Although citation rankings may differ among databases such as Scopus, PubMed, Embase, and Google Scholar, Web of Science is considered one of the most established and internationally recognized databases for citation-based scientific evaluation and was therefore considered appropriate for assessing global publication and citation trends in home mechanical ventilation research. The search strategy, screening approach, and eligibility criteria were predefined before data extraction; however, no formal preregistered protocol was used.

Total citations and citations per year were extracted directly from WoS at the time of data retrieval. Annual citation values were calculated by WoS and recorded as reported in the database (Table 1).

### 2.3. Search Timing and Scope After Ethics Approval

Following approval by the University Non-Interventional Clinical Research Ethics Committee the bibliometric search and data extraction were conducted (1 February 2026). The search was designed to identify influential publications addressing home mechanical ventilation, with particular emphasis on invasive long-term ventilatory support in the home setting. Clinically relevant invasive HMV was operationally defined as home-based ventilatory support delivered through tracheostomy or other invasive interface, or as mixed HMV service/programme, cohort, technical, caregiver, or policy studies in which IMV users were explicitly included or the clinical question directly concerned long-term ventilator-assisted home care with potential IMV involvement. Studies including mixed HMV populations involving both IMV and non-invasive ventilation (NIV) were eligible under this definition. Studies focusing exclusively on NIV without a direct relationship to invasive HMV, IMV transition, or mixed HMV service delivery were excluded.

### 2.4. Eligibility Criteria

Inclusion criteria were as follows:

- Indexed in Web of Science.
- Directly related to home mechanical ventilation.
- Ranked among the top 50 publications according to total citation count.

All article types (original articles, reviews, editorials, letters, guidelines, case reports, and other formats) were eligible for inclusion.

### 2.5. Data Extraction

For each of the 50 publications, the following variables were extracted and recorded:

- Total citations (WoS).
- Citations per year (WoS).
- Publication year (categorized as <2000, 2000–2004, 2005–2009, 2010–2014, 2015–2019, >2020).
- Journal name.
- Journal country.
- Journal continent.
- Journal quartile (Q index: Q1–Q4 or other).
- Journal impact factor and 5-year impact factor (when available).
- Publisher country and continent.
- Corresponding author continent.
- Multi-country publications were analyzed according to the corresponding author affiliation using a full-counting approach.
- Article type (article, review, editorial, letter, guideline).
- Binary article type classification (article vs. other).
- Topic category (as defined in Table 2).
- Funding status: Funding status and funding provenance were extracted from the Web of Science funding-related metadata fields, including available funding agency and funding text information. Records with identifiable funding information in Web of Science were classified according to the reported source as government/public, industry, philanthropic/non-profit, mixed, or not reported. Because only 8 publications had funding information recorded in Web of Science and 42 records had no identifiable funding information in these metadata fields, funding status was treated as a descriptive variable only. Absence of funding information in Web of Science was not interpreted as evidence that a study was unfunded, and no reliable inferential conclusion was drawn from comparisons by funding status.
- Open access status (yes/no).
- Index status (SCI-E vs. non–SCI-E/discontinued/book-series/ESCI).
- Number of references.
- Number of pages.

Topic categories were classified according to thematic content as shown in Table 2 (e.g., clinical outcomes & survival, healthcare organization/home care models, technical issues/ventilator malfunction/safety, caregiver burden/psychosocial impact, and others). Topic assignment was performed according to the primary thematic focus of each publication based on title, abstract, and full-text evaluation. Classification and screening procedures were conducted independently by both investigators (F.D.A. and V.H.). Inter-reviewer agreement was evaluated using Cohen’s kappa analysis and demonstrated excellent agreement (κ = 0.876, *p* < 0.001). Disagreements regarding study inclusion or topic categorization were resolved through consensus discussion, and the final dataset reflects the consensus-based classification. Journal quartile (Q index) classification was based on Journal Citation Reports data corresponding to the publication year when available. If quartile information was not available, it was categorized as “other.”

The complete rank-ordered list of the 50 publications, including first author, title, journal, PubMed ID, total citations, and citations per year, is presented in Table 1.

### 2.6. Ethics Statement

Ethical approval for this bibliometric study was obtained from the Canakkale Onsekiz Mart University Non-Interventional Clinical Research Ethics Committee (Meeting date: 28 January 2026; Meeting number: 2026-02/02-11; Protocol number: 2026-03). The study was reviewed in terms of its rationale, objectives, methodological approach, and scientific design and was approved by majority vote of the committee members.

The project titled “Bibliometric Analysis of the Most-Cited Publications in the Field of Home Mechanical Ventilation” was deemed ethically and scientifically appropriate. The study was conducted in accordance with the principles outlined in the Declaration of Helsinki.

As the analysis was based solely on publicly available bibliometric data and did not involve human participants, informed consent was not required.

### 2.7. Statistical Analysis

All statistical analyses were performed using IBM SPSS Statistics for Windows, version 24.0 (IBM Corp., Armonk, NY, USA).

The distribution of continuous variables, including total citations and citations per year, was assessed using the Shapiro–Wilk and Kolmogorov–Smirnov tests. As citation data did not demonstrate normal distribution, non-parametric methods were applied throughout the analysis. Continuous variables are presented as median (minimum–maximum) values, consistent with Table 2.

Comparisons of total citations and citations per year across multiple independent groups (e.g., publication year groups, journal quartile [Q index], journal continent, publisher continent, topic categories, and index status) were conducted using the Kruskal–Wallis test. When a statistically significant overall difference was identified, pairwise subgroup comparisons were performed using the Mann–Whitney U test with Bonferroni correction to adjust for multiple testing.

For binary comparisons (e.g., open access status, article vs. other document types), the Mann–Whitney U test was used for continuous citation variables. Funding status was excluded from inferential binary comparison and was summarized descriptively because reporting was incomplete and potentially influenced by historical reporting practices. Categorical variables were analyzed using the chi-square test where appropriate.

The relationships between citation metrics (total citations and citations per year) and bibliometric characteristics (reference count, number of pages, journal impact factor, journal 5-year impact factor, and year of publication) were evaluated using Spearman rank correlation analysis, as shown in Table 3.

A two-tailed *p* value of <0.05 was considered statistically significant. Field-normalized citation metrics were not applied because the study was designed as a descriptive citation-based bibliometric analysis within a relatively specialized research field. Given the relatively limited sample size and small subgroup distributions in several comparisons, particularly in categories with very low cell counts, the statistical analyses should be interpreted as exploratory and descriptive rather than confirmatory. Therefore, statistically significant subgroup findings should be interpreted cautiously, as some estimates may be unstable because of sparse subgroup distributions.

## 3. Results

### 3.1. General Citation Characteristics

**Figure 2:**
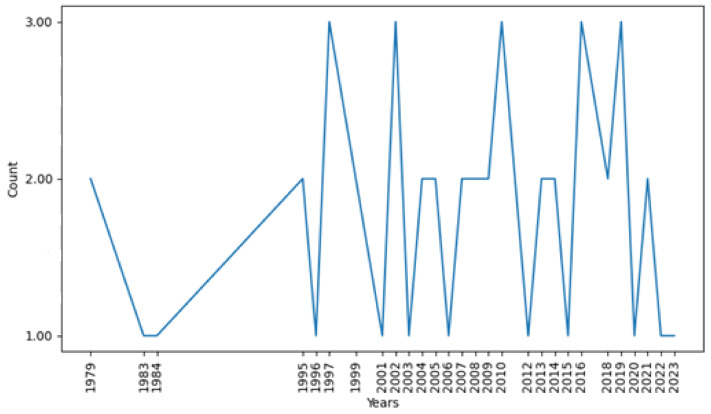
Distribution of the 50 most-cited articles on home mechanical ventilation by year. The figure shows the annual frequency of publications included in the bibliometric analysis.

**Figure 3:**
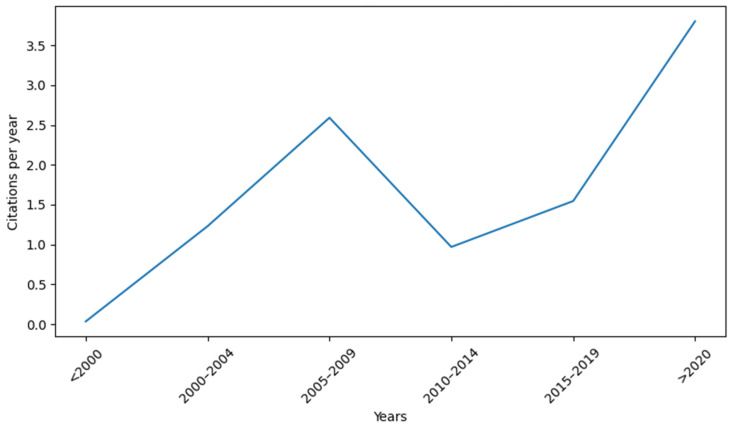
Change in median citations per year by publication year group. Median values were calculated for each predefined publication period (<2000, 2000–2004, 2005–2009, 2010–2014, 2015–2019, and >2020).

The 50 most-cited publications on home mechanical ventilation demonstrated a wide dispersion in citation performance. As shown in Table 2, median total citation counts and citations per year varied substantially across predefined subgroups. When grouped by publication year, statistically significant differences were observed for both total citations (*p* = 0.003) and citations per year (*p* < 0.001). Publications from 2005 to 2009 showed the highest median total citation count (56 [3,4,5,6,7,8,9,10,11,12,13,14,15,16,17,18,19,20,21,22,23,24,25,26,27,28,29,30,31,32,33,34,35,36,37,38,39,40,41,42,43,44,45,46,47,48,49,50,51,52,53,54,55,56,57,58,59,60,61,62,63,64,65,66]), whereas the most recent group (>2020) demonstrated the highest median citations per year (3.80 [1.00–6.43]). In contrast, articles published before 2000 had very low median total citations (1 [1,2,3,4,5,6,7,8,9,10,11,12,13,14,15,16,17,18,19,20,21,22,23,24,25]) and citations per year (0.04 [0.02–0.78]). The temporal distribution of included publications is illustrated in Figure 2, and the trend in median citations per year across publication periods is shown in Figure 3.

### 3.2. Article Type and Topic Distribution

Publications were classified as original articles (*n* = 36) or other document types (*n* = 14). Median total citation counts were 16.0 (range: 1–118) for original articles and 15.5 (range: 1–57) for other document types. Median citations per year were 0.89 (range: 0.02–9.83) and 0.92 (range: 0.02–6.43), respectively. No statistically significant differences were identified between the groups in terms of total citations (*p* = 0.803) or citations per year (*p* = 0.854).

**Figure 4:**
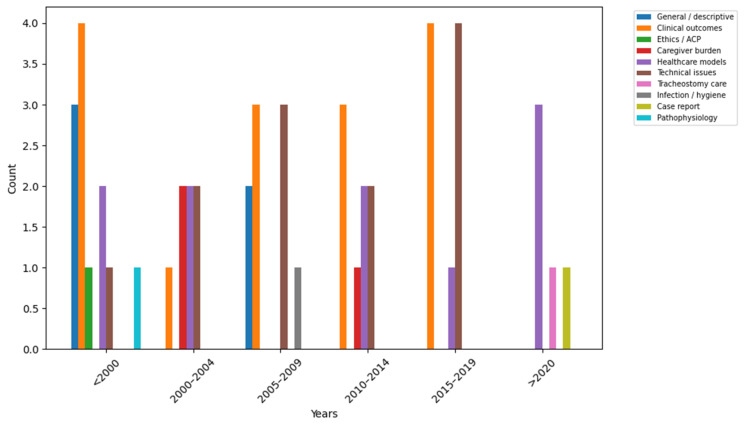
Distribution of article topics by publication year group. The grouped bar chart illustrates the frequency of different research topics across predefined publication periods.

**Figure 5:**
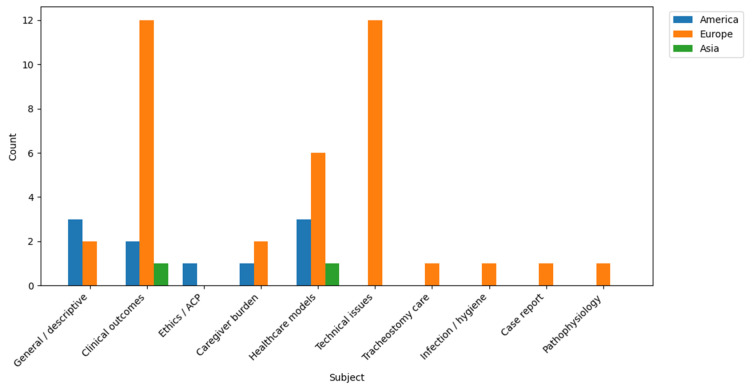
Distribution of research topics according to the continent of the corresponding author. The grouped bar chart presents the frequency of each topic across America, Europe, and Asia.

Topic categories were diverse, with the largest proportion addressing clinical outcomes & survival (*n* = 15), followed by technical issues/ventilator malfunction/safety (*n* = 12) and healthcare organization/home care models (*n* = 10). Median citation metrics varied across topic categories; however, no statistically significant differences were observed for total citations (*p* = 0.677) or citations per year (*p* = 0.620). The evolution of research themes over time is presented in Figure 4, and the geographic distribution of topics according to corresponding author continent is illustrated in Figure 5.

In the additional analysis performed after categorizing the included publications by ventilation population (IMV-only, NIV-only, or mixed/unspecified IMV/NIV), no statistically significant differences were observed in total citations or citations per year between ventilation population categories (*p* > 0.05).

**Figure 6:**
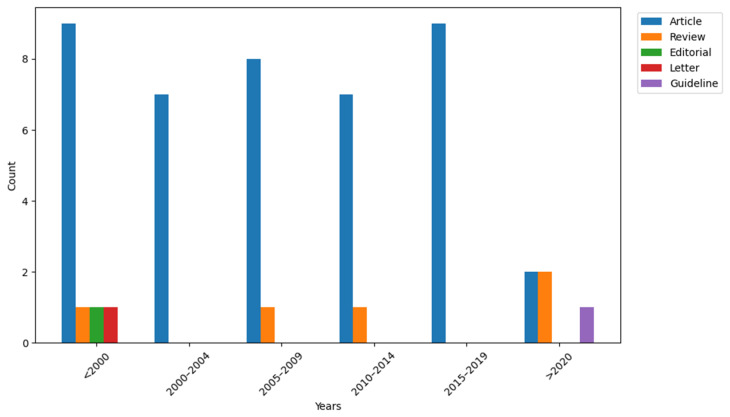
Distribution of article types according to publication year group. The grouped bar chart shows the frequency of original articles, reviews, editorials, letters, and guidelines across predefined publication periods.

The distribution of article types across publication periods is summarized in Figure 6.

### 3.3. Journal Characteristics and Geographic Distribution

Most articles were published in European (*n* = 33) and American (*n* = 16) journals. No statistically significant differences were observed according to journal continent (total citations: *p* = 0.308; citations per year: *p* = 0.329). Similarly, journal country was not significantly associated with citation metrics (total citations: *p* = 0.651; citations per year: *p* = 0.392). Journal quartile (Q index) showed an exploratory association with citation performance. Publications in Q1 journals (*n* = 18) had a median total citation count of 28 (1–66) and median citations per year of 2.13 (0.02–6.43). In contrast, Q4 journals (*n* = 7) showed lower medians for both total citations (1 [1,2,3,4,5]) and citations per year (0.03 [0.02–0.20]). Differences across quartile groups were statistically significant for both total citations and citations per year (*p* < 0.001 for both), but these subgroup findings should be interpreted cautiously.

### 3.4. Index Status, Funding, and Open Access

SCI-E indexed publications (*n* = 42) achieved significantly higher citation metrics compared with non–SCI-E/discontinued/book-series/ESCI publications (*n* = 8). Median total citations were 19 (1–118) versus 1 (1–16), and median citations per year were 1.29 (0.02–9.83) versus 0.03 (0.02–0.50), respectively (*p* < 0.001 for both comparisons). According to Web of Science funding-related metadata, funding information was available for 8 publications, whereas 42 records had no identifiable funding information in the Web of Science funding fields. Among the publications with available funding metadata, reported sources included public/governmental, institutional, and non-profit support where specified. Because funding-reporting practices may have differed across publication periods, particularly among older studies, records without Web of Science funding information were classified as “funding not reported” rather than definitively non-funded. Funding status was therefore presented descriptively only; no reliable conclusion can be made regarding the relationship between funding and citation impact in this dataset.

Open access status did not significantly influence total citations (*p* = 0.366) or citations per year (*p* = 0.897).

### 3.5. Corresponding Author and Publisher Characteristics

Most corresponding authors were based in Europe (*n* = 33), followed by American journals (*n* = 16). No statistically significant differences were observed in citation metrics according to corresponding author continent. Similarly, publisher country and publisher continent were not significantly associated with differences in total citations or citations per year (*p* > 0.05 for all comparisons).

### 3.6. Correlation Analysis

Spearman correlation analysis results are presented in Table 3. Total number of citations demonstrated a moderate positive correlation with number of pages (r = 0.539, *p* < 0.01), journal impact factor (r = 0.443, *p* < 0.01), and journal 5-year impact factor (r = 0.527, *p* < 0.01). No meaningful correlation was observed between total citations and reference count (r = −0.006). The correlation between total citations and year of publication was weak (r = 0.256). Citations per year showed significant positive correlations with number of pages (r = 0.609, *p* < 0.01), journal impact factor (r = 0.416, *p* < 0.01), journal 5-year impact factor (r = 0.510, *p* < 0.01), and year of publication (r = 0.548, *p* < 0.01). The association with reference count was not statistically significant (r = 0.128).

### 3.7. Ranking of the Top Publications

The detailed rank-ordered list of the 50 publications, including first author, title, journal, PubMed ID, total citations, and citations per year, is presented in Table 1. The most cited study was “Home Mechanical Ventilation in Canada: A National Survey” (118 total citations; 9.83 citations per year), followed by studies addressing air leaks in neuromuscular disorders and limitations of transcutaneous carbon dioxide monitoring.

## 4. Discussion

This bibliometric analysis of the 50 most-cited publications on home mechanical ventilation provides a structured overview of citation dynamics, thematic distribution, and journal-related patterns of scientific visibility in this field. Several findings merit detailed consideration.

### 4.1. Temporal Dynamics and Citation Acceleration

A temporal pattern was observed. Although earlier publications (particularly those from 2005 to 2009) demonstrated higher median total citation counts, more recent publications—especially those published after 2020—showed higher citations per year [3,10]. The exploratory association between publication year and citations per year, supported by Spearman correlation analysis (r = 0.548, *p* < 0.01; Table 3), may indicate an acceleration of citation intensity in recent years.

This pattern likely reflects multiple interacting factors. First, the expansion of digital dissemination and indexing platforms facilitates faster citation cycles. Second, increasing global awareness of chronic respiratory care, aging populations, and home-based care models may have amplified the clinical relevance of HMV research. Third, newer studies benefit from larger, more interconnected research networks, potentially enhancing citation propagation. Importantly, the discrepancy between total citations and citations per year underscores the dynamic nature of bibliometric evaluation: older articles accumulate citations over time, whereas newer publications may demonstrate higher annual impact despite lower cumulative counts [3,60].

### 4.2. Journal Quality and Index Status

Exploratory journal-related patterns were observed in citation performance. Publications in Q1 journals showed higher median total citations and citations per year compared with lower quartiles (*p* < 0.001 for both; Table 2). Furthermore, positive correlations were observed between citation metrics and journal impact factor as well as 5-year impact factor.

These findings are consistent with broader bibliometric literature suggesting that journal prestige, visibility, and distribution networks contribute to enhanced citation exposure [3,60]. Q1 journals typically possess higher readership, broader international reach, and greater indexing penetration, which collectively facilitate citation accrual [61]. Similarly, SCI-E-indexed publications significantly outperformed non-SCI-E/discontinued/book-series/ESCI publications in both total citations and citations per year (*p* < 0.001). This observation highlights the structural influence of indexing systems on scholarly dissemination.

Interestingly, journal country and continent did not significantly influence citation outcomes. This suggests that journal impact metrics and index status may exert greater influence than geographic location alone.

### 4.3. Funding and Open Access

Funding information was available in the Web of Science funding-related metadata for only 8 of the 50 included publications, whereas 42 records had no identifiable funding information in these metadata fields. Accordingly, funding status was treated as a descriptive variable rather than an inferential factor. Although median citation values differed between publications with available Web of Science funding information and those without recorded funding information, no reliable conclusion can be made about the relationship between funding and citation impact in this dataset. The apparent difference may reflect historical reporting practices, article age, indexing status, and the absence or incompleteness of structured funding metadata in older publications rather than a true funding effect. We therefore avoided interpreting funding status as a determinant of citation performance. Future bibliometric studies could improve this assessment by using grant-to-publication linkage, systematic acknowledgment-text mining, and sensitivity analyses based on plausible funding distributions.

Open access status did not significantly affect citation metrics in this dataset. While open access is frequently associated with improved visibility, its influence may be less pronounced in highly specialized clinical domains where readership is concentrated within professional networks [62,63,64,65].

### 4.4. Thematic Structure of Highly Cited Research

The most represented topics included clinical outcomes & survival, technical issues/ventilator malfunction/safety, and healthcare organization/home care models. These findings reflect the dual clinical–organizational nature of HMV [66]. Influential studies have addressed not only survival and physiological monitoring but also system-level challenges such as equipment quality control, service delivery models, and caregiver perspectives [5,7]. Several highly cited publications also contributed substantially to the scientific and technical evolution of home mechanical ventilation practices. Earlier influential studies focused predominantly on survival outcomes, ventilator monitoring, chronic respiratory failure management, and tracheostomy-related care in neuromuscular diseases. Over time, the literature increasingly expanded toward multidisciplinary home care models, caregiver burden, quality-of-life assessment, telemonitoring strategies, and healthcare system organization. In addition, technical investigations evaluating ventilator performance, air leaks, humidification systems, and equipment safety contributed to improving the reliability and effectiveness of long-term home ventilatory support.

Notably, no statistically significant differences in citation metrics were observed across topic categories. This suggests that influence in the HMV field is distributed across multiple thematic domains rather than concentrated in a single research focus [7,14].

The distribution of topics over time (Figure 3) indicates an evolution from earlier technical and survival-oriented studies toward broader organizational and health system perspectives [5,7,14]. Similarly, Figure 4 provides a descriptive overview of the geographic distribution of corresponding authors within the indexed literature analyzed in the present study, with European contributions being numerically predominant. However, these findings should be interpreted cautiously because database coverage, language representation, national publication incentives, and indexing practices may differ across countries and regions. Therefore, the observed geographic patterns may not fully reflect actual global research productivity or healthcare system activity in home mechanical ventilation.

### 4.5. Article Type and Structural Characteristics

Original research articles constituted the majority of publications, and no significant citation differences were observed between article types. This contrasts with some medical specialties where guidelines or consensus statements dominate citation rankings [2,3]. In the HMV field, impactful contributions appear to arise from diverse formats, including surveys, cohort studies, technical evaluations, and consensus documents [5,7].

Correlation analysis further revealed moderate positive associations between citation metrics and number of pages, journal impact factor, and 5-year impact factor (Table 3). The association with number of pages may reflect the comprehensive nature of influential studies, which often present detailed methodological or technical analyses.

### 4.6. Interpretation Within the Broader Bibliometric Context

The patterns observed align with established bibliometric principles across medical disciplines [1,3,10]. Publication recency may influence annual citation velocity; journal prestige may enhance visibility; and indexing status may amplify dissemination [67]. However, certain findings—particularly the absence of an open access effect and the incomplete reporting of funding information—should be interpreted descriptively because citation patterns in the HMV field may also reflect foundational clinical relevance, article age, and historical publication practices.

Home mechanical ventilation represents a specialized yet multidisciplinary field, intersecting pulmonology, neurology, rehabilitation medicine, intensive care, and health services research [9]. Compared with broader biomedical specialties, home mechanical ventilation research has a relatively smaller publication ecosystem and narrower citation network, which may contribute to lower absolute citation counts even among influential publications. The diversity of highly cited topics underscores this multidimensional character. The most influential publications frequently address real-world clinical challenges—such as ventilator leaks, monitoring limitations, national service models, and quality control—which may explain their sustained citation relevance.

Overall, exploratory associations in this HMV dataset were observed for publication year, journal quartile (Q index), index status, and journal impact metrics, whereas geographic origin, article format, open access status, and funding-reporting status should be interpreted descriptively. These findings provide a structured overview of citation patterns in this domain and may inform future research dissemination strategies, but they should not be interpreted as firm determinants of scholarly influence. Cross-country comparisons in bibliometric analyses are inherently influenced by database coverage, language bias, national publication incentives, and field-specific indexing practices. Consequently, geographic publication patterns observed in the present study should be interpreted descriptively rather than as definitive indicators of national scientific productivity or healthcare system performance. In addition, bibliometric indicators alone may not fully capture the real-world clinical, organizational, or policy impact of research activity. Future investigations integrating citation-based analyses with complementary indicators such as clinical guideline citations, major grant activity, policy implementation measures, clinical trial registrations, patent activity, and workforce or training metrics may provide a more comprehensive understanding of translational impact in home mechanical ventilation research.

Several limitations of this study should be acknowledged. First, the analysis was based solely on the Web of Science Core Collection database, and citation rankings may differ across other bibliographic platforms such as Scopus, PubMed, or Google Scholar. Second, the study focused on the 50 most-cited publications within a relatively specialized clinical field, which may limit generalizability of the findings. In addition, several subgroup analyses included very small category sizes, including some groups with single-study representation, which may have limited the stability and interpretability of certain statistical comparisons. Consequently, subgroup-related findings should be considered exploratory and hypothesis-generating rather than definitive. Third, citation-based metrics are influenced by publication age, indexing status, journal visibility, and historical citation practices. Fourth, funding status was based on Web of Science funding-related metadata rather than systematic full-text verification of every article. Therefore, the 42 records without identifiable Web of Science funding information should be interpreted as “funding not reported in Web of Science” and not as definitively unfunded publications. Incomplete historical reporting of funding information, especially in older studies, may have affected descriptive funding patterns. Finally, the absence of field-normalized citation metrics and the use of bibliometric indicators alone may not fully reflect the clinical, organizational, educational, or policy impact of research activity in home mechanical ventilation.

## 5. Conclusions

This bibliometric analysis of the 50 most-cited publications on home mechanical ventilation observed exploratory associations between citation impact and publication year, journal quartile, index status, and journal impact metrics within the analyzed dataset. The included publications covered diverse topics related to long-term ventilatory care, including clinical outcomes, technical aspects, healthcare organization, and home care models.

This bibliometric analysis provides a descriptive overview of publication trends, citation characteristics, and journal-related patterns associated with scientific visibility in home mechanical ventilation research. Citation impact within the analyzed dataset showed exploratory associations with publication year, journal quartile, index status, and journal impact metrics. These findings contribute to understanding the academic development and evolving research patterns of the field and may help inform future bibliometric and research evaluation studies. However, the results should be interpreted within the methodological limits of a 50-publication bibliometric analysis and should not be considered confirmatory determinants of citation impact or direct indicators of clinical effectiveness, healthcare quality, or policy impact.

## Figures and Tables

**Table 1:** Information on the 50 most-cited studies included in our study.

Rank	First Author	Title	Journal	PubMed ID	Total Citation (WOS)	Annual Citation per Year (WOS)	Ventilation Population
1	Rose, L	Home Mechanical Ventilation in Canada: A National Survey [10]	Respiratory Care	25587173	118	9.83	Mixed IMV/NIV
2	Gonzalez, J	Air leaks during mechanical ventilation as a cause of persistent hypercapnia in neuromuscular disorders [11]	Intensive Care Medicine	12589533	75	3.12	Mixed IMV/NIV
3	Cuvelier A	Limitations of transcutaneous carbon dioxide measurements for assessing long-term mechanical ventilation [12]	Chest	15888854	66	3	Mixed IMV/NIV
4	Marchese S	Outcome and attitudes toward home tracheostomy ventilation of consecutive patients: A 10-year experience [13]	Respiratory Medicine	18023334	62	3	IMV-only
5	Farre R	Quality control of equipment in home mechanical ventilation: a European survey [14]	European Respiratory Journal	15994393	57	3	Mixed/unspecified IMV/NIV
6	Oliveira ASB	Amyotrophic lateral sclerosis (ALS) three letters that change the people’s life for ever [15]	Arquivos de Neuro-Psiquiatria	19722069	57	3	Mixed IMV/NIV
7	Laub M	Survival of patients on home mechanical ventilation: A nationwide prospective study [16]	Respiratory Medicine	17118638	56	3	Mixed/unspecified IMV/NIV
8	Tassaux D	Comparative bench study of triggering pressurization, and cycling between the home ventilator VPAP II and three ICU ventilators [17]	Intensive Care Medicine	12209273	54	2.25	Mixed IMV/NIV
9	Chatwin M	Analysis of home support and ventilator malfunction in 1211 ventilator-dependent patients [18]	European Respiratory Journal	19643945	45	3	Mixed IMV/NIV
10	Klingshirn H	Quality of Care for People with Home Mechanical Ventilation in Germany: A Scoping Review [19]	Gesundheitswesen	32650350	45	6	Mixed/unspecified IMV/NIV
11	Lewarski JS	Current issues in home mechanical ventilation [20]	Chest	17699139	43	2	Mixed/unspecified IMV/NIV
12	Evans R	“Family caregiver perspectives on caring for ventilator-assisted individuals at home” [21]	Canadian Respiratory Journal	23248801	39	3	IMV-only
13	Janssens JP	“Long-Term Mechanical Ventilation: Recommendations of the Swiss Society of Pulmonology [22]	Respiration	33302274	38	6	Mixed IMV/NIV
14	van Kesteren RG	Psychosocial problems arising from home ventilation [23]	American Journal of Physical Medicine & Rehabilitation	11399005	32	1	Mixed IMV/NIV
15	Stuart M	Integrated health system for chronic disease management—Lessons learned from France [24]	Chest	14769754	31	1	Mixed/unspecified IMV/NIV
16	Divo MJ	Prolonged Mechanical Ventilation in Massachusetts: The 2006 Prevalence Survey [25]	Respiratory Care	21122178	29	2	Mixed/unspecified IMV/NIV
17	Raphaël JC	Assessment of quality of life for home ventilated patients with Duchenne muscular dystrophy [26]	Revue Neurologique	11984488	27	1	Mixed IMV/NIV
18	Goldstein RS	Home Mechanical Ventilation: Demographics and User Perspectives [27]	Chest	7497765	25	1	Mixed IMV/NIV
19	Bonnici DM	Prospective observational cohort study of patients with weaning failure admitted to a specialist weaning, rehabilitation and home mechanical ventilation centre [28]	BMJ Open	26956162	25	2	Mixed IMV/NIV
20	Boussaïd G	Effect and impact of mechanical ventilation in myotonic dystrophy type 1: a prospective cohort study [29]	Thorax	29572271	19	2	NIV-only
21	Haziot N	Impact of leaks and ventilation parameters on the efficacy of humidifiers during home ventilation for tracheostomized patients: a bench study [30]	BMC Pulmonary Medicine	30777036	19	2	IMV-only
22	Toussaint M	Building a home ventilation programme: population, equipment, delivery and cost [31]	Thorax	35868847	19	4	Mixed/unspecified IMV/NIV
23	Janssens JP	Validity and reliability of a French version of the MRF-28 health-related quality of life questionnaire [32]	Respiration	15627866	18	1	Mixed/unspecified IMV/NIV
24	Hannan LM	Care Practices and Health-related Quality of Life for Individuals Receiving Assisted Ventilation [33]	Annals of the American Thoracic Society	27295155	17	2	Mixed IMV/NIV
25	CRIEE CP	Respirator Dependency—Intensive-Care Unit or Home Ventilation Unit [34]	Internist	7558700	16	1	Mixed/unspecified IMV/NIV
26	Rose L	Patient transitions relevant to individuals requiring ongoing ventilatory assistance: A Delphi study [35]	Canadian Respiratory Journal	24791254	15	1	Mixed/unspecified IMV/NIV
27	Winterholler M	Home ventilation of adults with neuromuscular diseases—Bavarian consensus [36]	Nervenarzt	9273467	15	1	Mixed/unspecified IMV/NIV
28	Gamez J	Cellular transplants in amyotrophic lateral sclerosis patients: an observational study [37]	Cytotherapy	20586670	12	1	Mixed IMV/NIV
29	Raaphorst J	Treatment of respiratory impairment in patients with motor neuron disease in the Netherlands: patient preference and timing of referral [38]	European Journal of Neurology	23398243	11	1	NIV-only
30	Orlikowski D	Comparison of ventilator-integrated end-tidal CO_2_ and transcutaneous CO_2_ monitoring in home-ventilated neuromuscular patients [39]	Respiratory Medicine	27492508	8	1	Mixed IMV/NIV
31	Toussaint M	Is disinfection of mechanical ventilation tubing needed at home? [40]	International Journal of Hygiene and Environmental Health	16376145	7	0.33	Mixed IMV/NIV
32	Summ O	Central Bradypnea and Ataxic Breathing in Myotonic Dystrophy Type 1-A Clinical Case Report [41]	Journal Of Neuromuscular Diseases	36911946	7	2	NIV-only
33	Sancho J	Mechanical Insufflation-Exsufflation With Oscillations in Amyotrophic Lateral Sclerosis With Home Ventilation via Tracheostomy [42]	Respiratory Care	33082217	6	1	IMV-only
34	Tan GP	The pattern of use and survival outcomes of a dedicated adult Home Ventilation and Respiratory Support Service in Singapore: a 7-year retrospective observational cohort study [43]	Journal of Thoracic Disease	31019767	6	1	Mixed IMV/NIV
35	Clemente F	Critical failures in the use of home ventilation medical equipment [44]	Heliyon		6	1	Mixed/unspecified IMV/NIV
36	Stieglitz S	Frequency and management of respiratory incidents in invasive home ventilation [45]	Chronic Respiratory Disease	23897929	6	0.40	IMV-only
37	Guber A	First experience with the home-care management system for respiratory patients in Israel [46]	Israel Medical Association Journal	12073412	5	0.20	Mixed/unspecified IMV/NIV
38	Mikesch M	Hom invasive and non-invasive ventilation in severe COPD [47]	Wiener Medizinische Wochenschrift	20151349	5	0.20	Mixed IMV/NIV
39	LOH, L	Home Ventilation [48]	Anaesthesia	6869734	5	0.10	Mixed/unspecified IMV/NIV
40	De Mattia	Passive Versus Active Circuit During Invasive Mechanical Ventilation in Subjects With Amyotrophic Lateral Sclerosis [49]	Respiratory Care	29765003	4	0.40	IMV-only
41	Quinlivan R	Innovative care model for patients with complex muscle diseases [50]	Current Opinion In Neurology	25188015	4	0.30	Mixed/unspecified IMV/NIV
42	Wiebel M	IPPV for respiratory failure due to restrictive disorders of the chest wall [51]	Medizinische Klinik	8684317	3	0.10	IMV-only
43	Chatwin M	Analysis of emergency helpline support for home ventilator dependent patients [52]	European Respiratory Review	18474664	3	0.20	Mixed/unspecified IMV/NIV
44	INDIHAR FJ	Home Ventilation [53]	Chest		1	0	Mixed/unspecified IMV/NIV
45	LARENG L	Study of home ventilation in severe respiratory insufficient patients [54]	Revue Francaise Des Maladies Respiratoires	398558	1	0	Mixed IMV/NIV
46	CARDINAUD JP	Problems raised by the technique and supervision of home ventilation of chronic respiratory insufficient patients [55]	Revue Francaise Des Maladies Respiratoires		1	0	Mixed/unspecified IMV/NIV
47	Raffenberg M	Invasive and non-invasive home ventilation—Changes between 1982 and 1996 [56]	Medizinische Klinik	10373729	1	0	Mixed IMV/NIV
48	Laier-Groeneveld G	Aetiology of chronic hypercapnic ventilatory failure [57]	Medizinische Klinik		1	0	Mixed/unspecified IMV/NIV
49	Winterholler M	Brain syndrome and home ventilation—Diagnosis, therapy and consequences [58]	Medizinische Klinik	10373740	1	0	Mixed/unspecified IMV/NIV
50	Wiebel M	Course of IPPV: Causes of mortality [59]	Medizinische Klinik	9235478	1	0	IMV-only

Note: Ventilation population was categorized as IMV-only, NIV-only, or mixed/unspecified IMV/NIV based on the study title, abstract, full-text information when available, and the described target population or service model. Mixed/unspecified IMV/NIV denotes studies that explicitly included both invasive and non-invasive ventilation or addressed HMV services, equipment, technical issues, or care models in which the ventilation interface was not restricted to one mode. Data are presented as rank-ordered publications by total citation count. Total citations and annual citations were obtained from Web of Science (WoS). Journal impact factor and quartile (Q1–Q4) refer to the Journal Citation Reports classification for the corresponding year (when available). Abbreviations: HMV, home mechanical ventilation; WoS, Web of Science; IF, impact factor; ACP, advance care planning; NIV, non-invasive ventilation; IMV, invasive mechanical ventilation. Annual citation values were calculated from Web of Science citation data and are presented with two-decimal precision for consistency. Subgroups with *n* < 5 were retained for descriptive presentation purposes only and were excluded from comparative statistical analyses.

**Table 2:** Characteristics of the 50 most-cited articles on home mechanical ventilation.

	Subgroups	N	Total Number of Citations Median (Min–Max)	Citations per Year Median (Min–Max)	*p* Value for Total Citations	*p* Value for Citations per Year
Publication year groups	<2000	12	1 (1–25)	0.04 (0.02–0.78)	0.003	<0.001
	2000–2004	7	31 (5–75)	1.23 (0.20–3.13)		
	2005–2009	9	56 (3–66)	2.59 (0.16–3.26)		
	2010–2014	8	13.5 (4–45)	0.97 (0.31–2.65)		
	2015–2019	9	17 (4–118)	1.55 (0.44–9.83)		
	>2020	5	19 (6–45)	3.80 (1.00–6.43)		
Journal quartile	Q1	18	28 (1–66)	2.13 (0.02–6.43)	<0.001	<0.001
	Q2	15	12 (3–118)	0.78 (0.11–9.83)		
	Q3	5	32 (6–57)	1.23 (0.50–3.17)		
	Q4	7	1 (1–5)	0.03 (0.02–0.20)		
	Other	5	6 (3–16)	0.43 (0.10–1.75)		
Funding	Funding not reported	42	19 (1–118)	1.19 (0.02–9.83)	Descriptive only	Descriptive only
	Funding reported	8	2 (1–7)	0.07 (0.03–1.75)		
Index	SCI-E	42	19 (1–118)	1.29 (0.02–9.83)	<0.001	<0.001
	Non–SCI-E/discontinued/book-series/ESCI	8	1 (1–16)	0.03 (0.02–0.50)		
Open access	No	39	17 (1–118)	0.79 (0.02–9.83)	0.366	0.897
	Yes	11	8 (1–39)	1.15 (0.02–2.60)		

Note: Funding status is reported descriptively only because only 8 publications contained identifiable funding information in Web of Science funding-related metadata fields and 42 records had no recorded funding information in these fields. Records without Web of Science funding information were not considered definitively non-funded, and no reliable inferential conclusion can be drawn from this comparison. Abbreviations: yr, year; ACP, advance care planning; SCI-E, Science Citation Index Expanded; ESCI, Emerging Sources Citation Index; Q, journal quartile; USA, United States of America; UK, United Kingdom. *p* values represent comparisons between subgroup distributions using the Kruskal–Wallis or Mann–Whitney U tests where appropriate.

**Table 3:** Correlation relationships and Spearman correlation coefficients between citation metrics and bibliometric characteristics of the included publications (*n* = 50).

	Reference Count	Number of Pages	Journal İmpact Factor	Journal 5-Year İmpact Factor	Year of Publication
Total number of citations	−0.006	0.539	0.443	0.527	0.256
Citations per year	0.128	0.609	0.416	0.510	0.548

Abbreviations: Reference count = number of references cited in the article. *p* < 0.05, Spearman correlation analysis. *p* < 0.01, Spearman correlation analysis. Citation metrics are presented with consistent decimal precision throughout the table.

## Data Availability

The raw data supporting the conclusions of this article will be made available by the authors on request.
